# The role of *ATP-binding cassette transporter* genes expression in treatment failure cutaneous leishmaniasis

**DOI:** 10.1186/s13568-022-01419-5

**Published:** 2022-06-16

**Authors:** Mohammad Javad Boozhmehrani, Gilda Eslami, Ali Khamesipour, Abbas Ali Jafari, Mahmood Vakili, Saeedeh Sadat Hosseini, Vahideh Askari

**Affiliations:** 1 Department of Parasitology and Mycology, Faculty of Medicine, Shahid Sadoughi University of Medical Sciences, Yazd, Iran; 2 Research Center for Food Hygiene and Safety, Shahid Sadoughi University of Medical Sciences, Yazd, Iran; 3 Center for Research and Training in Skin Diseases and Leprosy, Tehran University of Medical Sciences, Tehran, Iran; 4 Health Monitoring Research Center, School of Medicine, Shahid Sadoughi University of Medical Sciences, Yazd, Iran

**Keywords:** *Leishmania major*, Treatment failure, ATP-binding cassette transporters, Leishmaniasis, Cutaneous

## Abstract

Leishmaniasis is one of the common diseases transmitted by sand flies in tropical and subtropical regions of the world. Currently, antimonial derivatives are the first line of treatment. Some of the members of the ATP-binding cassette (ABC) family of *Leishmania* are shown to be associated with no response to treatment. In this study, we evaluated *ABCI4*, *ABCG2, ABCC7, ABCB4*, and *ABCC3* genes expression in *Leishmania* isolated from patients with non-healing cutaneous leishmaniasis and treatment response isolates. We selected 17 clinical isolates including 8 treatment failure and 9 treatment response samples from September 2020 to March 2021. The isolates were obtained from patients of Health Center Laboratory of Varzaneh, Isfahan, Iran with cutaneous leishmaniasis. The diagnosis was performed using microscopic observation. The samples were directly collected from the lesions. The expression profiling of genes was assessed using SYBR Green real-time PCR that was analyzed with delta-delta Ct. All treatment failure clinical isolates were *L. major*. Gene expression analysis in treatment failure isolates showed that the ABC transported genes had a different pattern in each isolate. Treatment failure has been reported for cutaneous leishmaniasis worldwide. Knowledge of the molecular mechanisms of treatment failure could solve this problem. ABC transporter genes are considered controversial over the mechanisms of treatment failure outcomes. In this study, we showed that ABC transporter genes could be considered one of the important mechanisms.

## Introduction

Leishmaniasis is a tropical neglected parasitic disease caused by different *Leishmania* spp., which is transmitted by *Phlebotomus* spp. The main clinical manifestations are Cutaneous Leishmaniasis (CL), kala-azar or Visceral Leishmaniasis (VL), and MucoCutaneous Leishmaniasis (MCL) (Alvar et al. 2012). Leishmaniasis is endemic in more than 100 countries with around 1 billion people at the risk of disease. WHO estimated 30 000 new cases of VL and more than 1 million new cases of CL annually (WHO 2020). Almost 80% of CL incidence, in 2020, is reported from Afghanistan, Albania, Algeria, Brazil, Colombia, Iran, Iraq, Pakistan, and the Syrian Arab Republic, etc. (WHO 2020). In Iran, CL is reported about 20 000 cases annually although it seems that the real cases would be higher. Both rural and urban CL is reported in Iran. The rural type is endemic in almost 15 provinces (Norouzinezhad et al. 2016).

Although CL is a self-limiting disease within a year, there are some cases with diffusion condition resulting in severe disease (Firooz et al. 2020). CL mostly appeared in the upper limbs, especially in the face, therefore it can harbor psychosocial burden in patients (Bennis et al. 2018). Hence, prevention of the disease is very important. No effective vaccine is developed against leishmaniasis so far, so treatment is considered as the most important solution to control the disease. The first line of treatment is antimonial derivatives including meglumine antimonate (Glucantime) and sodium stibogluconate (Pentostam) used for more than 80 years (Haldar et al. 2011). The recent reports show no response to drug and treatment failure clinical isolates from different parts of the world (Kakooei et al. 2020; Ponte-Sucre et al. 2017; Somee et al. 2021). Based on our knowledge, there are various mechanisms involving drug responses that some of them have been known (Decuypere et al. 2012). One of the most important reported mechanisms is low expression of the *aquaglyceroporin* 1 gene (*AQP*1), resulting in reduced entry of antimony drugs into the parasite (Somee et al. 2021). Other reported mechanisms are considered pumping out the drug by ATP-binding cassette (ABC) transporters (Ponte-Sucre et al. 2017).

ABC transporters are one of the most prominent families with vital physiological functions. These proteins are found in various species, from prokaryotes to humans, which use ATP hydrolysis to exclude multiple compounds across the membranes. The ABC transporters have crucial role in no response to drug through two mechanisms including overexpression and mutation of ABC-carrying genes (Ouellette et al. 1998). In *Leishmania*, ABC protein is considered multidrug resistance protein (MRP) with a major role in metal resistance using thiol metabolism and drug efflux mechanisms (Croft et al. 2006). P-glycoprotein A (PGPA) is a member of ABC transporter family involved in no response to drug, especially antimoniate and arsenite.

The *Leishmania* genome contains 42 *ABC* genes ranging from *ABCA* to *ABCH* (Leprohon et al. 2006). Among the available genes, a few studies have reported a link between non-response to glucantime and *ABCC3*, *ABCC7*, *ABCG2*, *ABCB4*, and *ABCI4* genes in *L. major* (Leprohon et al. 2015; Ponte-Sucre et al. 2017). The ABCI4 transporter is located in the mitochondria and plasma membrane and is involved in transporting heavy metals in the parasites. The *ABCI4* gene is located on chromosome 33 (Manzano et al. 2014). The ABCG2 transporter is located in intracellular vesicles and in the plasma membrane encoded by *ABCG2* gene located on chromosome 6. This transporter pumps the conjugated thiol-antimony compound out of the amastigotes cell (Leprohon et al. 2006; Brochu et al. 2003; Perea et al. 2016). The ABCC7 (PRP1) transporter is located in intracellular vesicles, encoded by *ABCC7* on chromosome 31. The ABCC3 transporter (MRPA) is also situated in vesicles between the nucleus and the flagellar pocket encoded by *ABCC3* gene located on chromosome 23. ABCC7 and MRPA proteins are associated with tubulin vesicles and thus bind to exocytosis and endocytosis pathways (Coelho et al. 2007; Leprohon et al. 2009; Mukherjee et al. 2007). The *ABCB4* is also involved in no response to drug in *L. donovani* by efflux the drug (Rastrojo et al. 2018). These proteins seem to play essential roles in response to the drug; therefore, the aim of the current study is to assess *ABCI4*, *ABCG2*, *ABCC7*, *ABCB4*, and *ABCC3* genes expression in clinical isolates of *Leishmania* obtained from patients harboring CL lesions with treatment failure outcome after a period of standard treatment with Glucantime.

## Materials and methods

### Ethical statement

Each patient enrolled to this study completed by writing the informed consent. All methods were done based on principles of the declaration of Helsinki. The study was approved by the Ethics Committee of Shahid Sadoughi University of Medical Sciences, Yazd, Iran. (IR. SSU. MEDICINE. REC. 1399.194).

### Study area

**Fig. 1:**
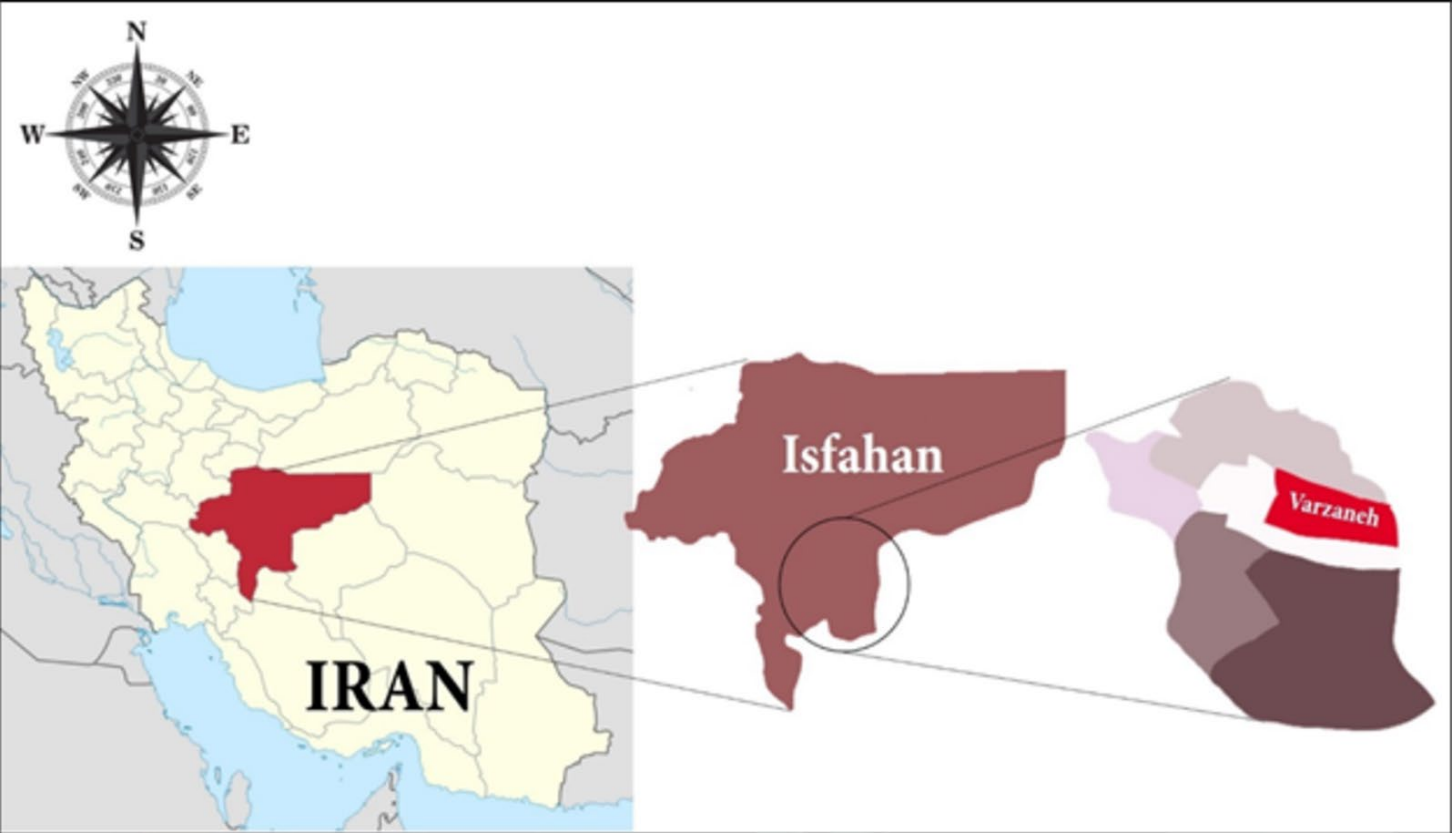
The geographical location of Varzaneh, Isfahan, Iran

Varzaneh is a city in the southeast of Isfahan province (Fig. 1). The city is located in the western part of Gavkhooni swamp, in longitude of 39′, 52° and latitude of 25′, 52° north, and is a well-known CL endemic area. The current population of the city is approximately 17,000.

### Clinical isolates

The clinical isolates of *Leishmania* were collected from patients of Health Center Laboratory of Varzaneh, Isfahan, Iran, from September 2020 to March 2021. The CL primary diagnosis was performed using direct smear and microscopic observation of Leishman body in Giemsa-stained slides. After diagnosis, the samples were directly collected from the lesion, transferred into RNA*later* solution (Merck, Darmstadt, Germany), and stored at − 20 °C for further analysis. Each CL case was administered with standard regimen treatment of glucantime (20 mg/kg/day for 20 days). Response to drug was evaluated by re-epithelialization of lesion and decreased inflamed border of the lesions at day 20 of drug administration. In cases of no response to drug, the additional administration period was applied. In no response cases, the patient was considered as treatment failure (TF) (Martínez et al. 2020; Vanaerschot et al. 2014) and the related isolate was focused for next experiments. The cases with the response to glucantime treatment were grouped as treatment response (TR).

### Verification and identification

The isolates were verified and identified using an Internal Transcribed Spacer (ITS)-1-PCR–RFLP (Eslami et al. 2016) using the specific primer pair of LITSR: 5′-CTTGGATCATTTTCCGATG-3′ and L5.8 S 5′-TGATACCACTTATCGCATT-3′ in a final concentration of 0.5 µM for each. The reaction was conducted using thermo cycler (SimpliAmp, ABI, USA) in a 20 µl reaction solution with the amplification condition described by Eslami et al. (2016) Positive and negative controls were run in each reaction using *L. major* (MRHO/IR/75/ER) and ddH_2_O, respectively. The amplification analysis was carried out using 1.5% agarose gel electrophoresis using gel documentation. The gel was stained using DNA Green Viewer (Pars Tous, Iran, Mashhad). The amplified fragment with the length from 300 to 350 bp was considered as *Leishmania.* In order to species identification, RFLP was performed using digestion of the amplicon by HaeIII restriction enzyme at 37 °C for 2 h. Digested fragments were assessed using 3% agarose gel electrophoresis stained with DNA Green Viewer alongside with 50 bp DNA ladder (CinnaGen, Iran, Tehran). The fragments with the length of 220 and 127 bp identified *L. major*; 220 and 50 bp recognized *L. tropica*; and 200, 100, and 50 bp known *L. infantum*.

### RNA extraction and cDNA synthesis

Total RNA was extracted using the total RNA extraction kit (Vivantis, Malaysia) based on description by the manufacturer. DNase I (CinnaGen, Iran, Tehran) was used to treat the extracted RNA to avoid any genomic contamination. The RNA quantity was estimated using Nanodrop (Thermo Fisher Scientific, USA). Then, complementary DNA (cDNA) was synthesized from total RNA (1 µg) using RevertAid First Strand cDNA Synthesis Kit (Thermo Fisher Scientific, USA) based on the manufacturer’s instructions. The primers used in this step were random hexamer and oligo (dT) primer.

### Gene expression analysis

The expression profiling of *ABCG2, ABCI4, ABCC7, ABCB4*, and *ABCC3* genes involving drug efflux were assessed using SYBR Green real-time PCR. The GAPDH gene (Eslami et al. 2016) was considered for normalization purposes, referred as internal or endogenous control. All primer pairs related to the *ABCG2, ABCI4, ABCC7, ABCB4*, and *ABCC3* were designed in this study (Table 1). Amplifications were done in a total volume of 20 µl containing 2 µl cDNA, 10 µl SYBR Green Real Time master mix (2X; ABI, USA), and primer pairs (with the final concentration shown in Table 1) using a Step One thermocycler (Applied Biosystem, USA). The thermal conditions of the reaction were 95 °C for 10 min in order to the first denaturation, followed by 40 cycles of 95 °C for 10 s and 60° C for 10 s. The specificity of the reaction products, no nonspecific products, and no primer dimers were confirmed by melting curve, which consisted of temperatures between 60 and 95 °C with a heating rate of 0.3 °C/s. All reactions were done in duplicate. In cases of necessary, the reactions were repeated in more duplicate set. The relative amount of amplification by each primer pair was determined based on threshold cycle (Ct) value of the interest gene, normalized to that of reference GAPDH gene. Gene expression analysis was done using 2^−ΔΔCT^ method using follow formula:

$$\Delta \Delta \text{CT}=\text{ }\left( \text{C}{{\text{T}}_{\text{target gene in sample}}}-\text{ C}{{\text{T}}_{\text{GAPDH in sample}}} \right)-\left( \text{C}{{\text{T}}_{\text{target gene in standard}}}-\text{ C}{{\text{T}}_{\text{GAPDH in standard}}} \right)$$

**Table 1:** The primer pairs used in this study for gene expressions of *ABCG2, ABCI4, ABCC7, ABCC3*, *ABCB4*, and GAPDH

Primer name	Sequence (5′-3′)	Target gene
ABCI4-F	TGCCGTCGTCTCGCATCTCTTTTCA	ABCI4
ABCI4-R	ACGGCAGCAGAGCGYAGAGAAAAGT	
ABCG2-F	TTCGCCGAGTTTCCCGTGCAGA	ABCG2
ABCG2-R	AAGCGGCTGAAAGGGCACGTCT	
ABCC7-F	AGGAGGGAGTGCGAAAAGGGCT	ABCC7
ABCC7-R	TCCTCCCTCACGCTTTTCCCGA	
ABCC3-F	AACATCTTTTGCTCCCCACTGCCC	ABCC3
ABCC3-R	TTGTAGAAAACGAGGGGTGACGGG	
ABCB4*-F*	AACCAACCTGTACGCTCCGCTGTTT	ABCB4
ABCB4*-R*	ATCCGTAAAAGCCGTGCAGAACCCA	
GAPDH-F	AGGACATTCTCGGCTTCACCAA	GAPDH
GAPDH-R	GCCCCACTCGTTGTCATACCA	

### Statistical analysis

Experiments were carried out in duplex and the data are presented as mean. Statistical comparisons between groups were performed using t-test. *P* value ≤ 0.05 was considered statistically significant. Statistical analyses were performed using SPSS version 26.0 (Chicago, IL).

## Results

**Fig. 2:**
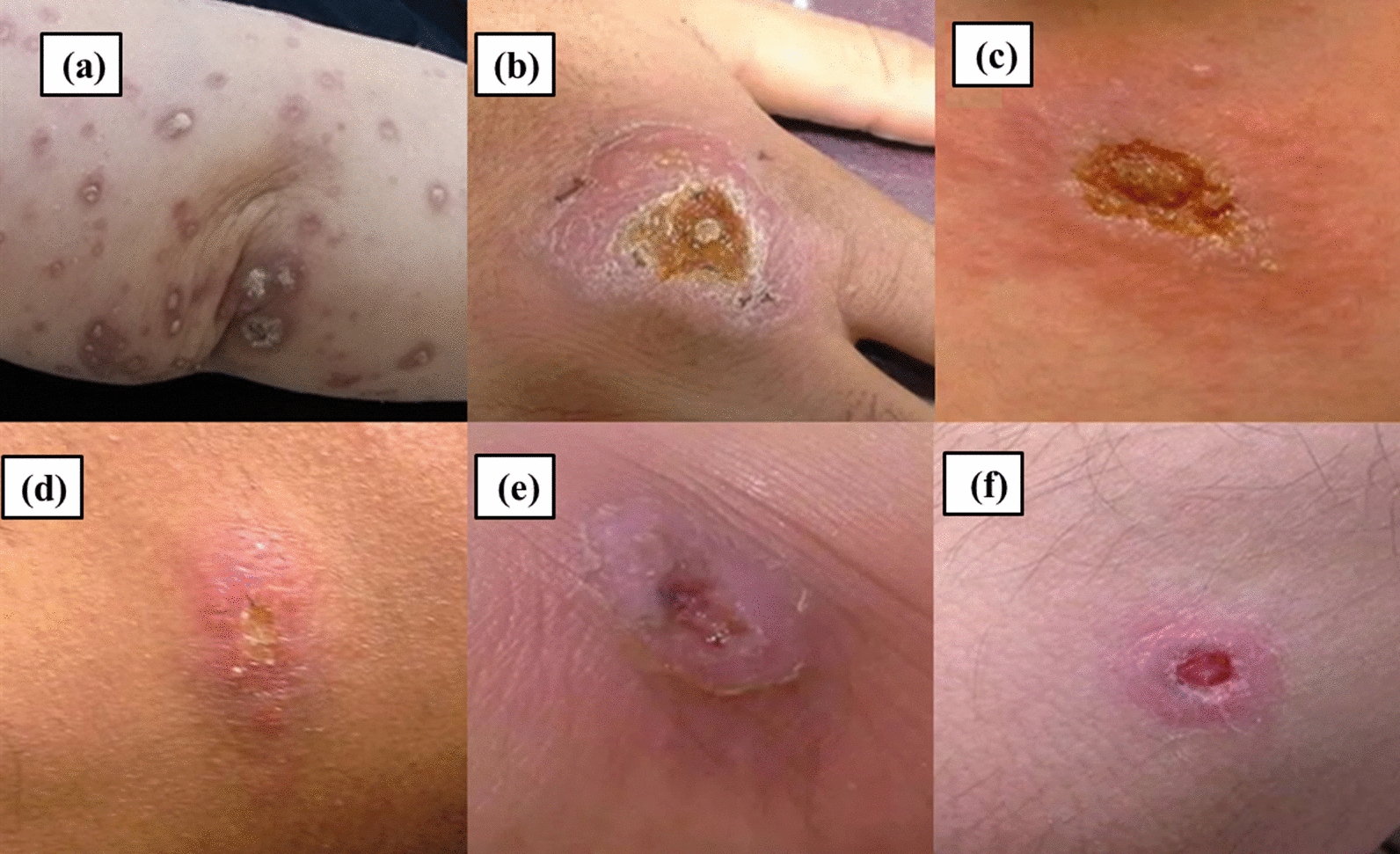
The lesion of cutaneous leishmaniasis from the patients. **a–c** Treatment failure patients; **d–f** treatment response patients

The mean ± SD of lesions’ size in patients with treatment failure was 5.4 ± 3 × 6.6 ± 1.85 cm^2^ and that in patients with treatment response was 4.33 ± 1.24 × 3 ± 1.44 cm^2^ (Fig. 2). The samples with treatment failure outcomes were encoded as TF1, TF2, TF3, TF4, TF5, TF6, TF7, and TF8; the isolates from treatment response were known as TR1, TR2, TR3, TR4, TR5, TR6, TR7, TR8, and TR9. TR1 was considered as the standard sample.

**Fig. 3:**
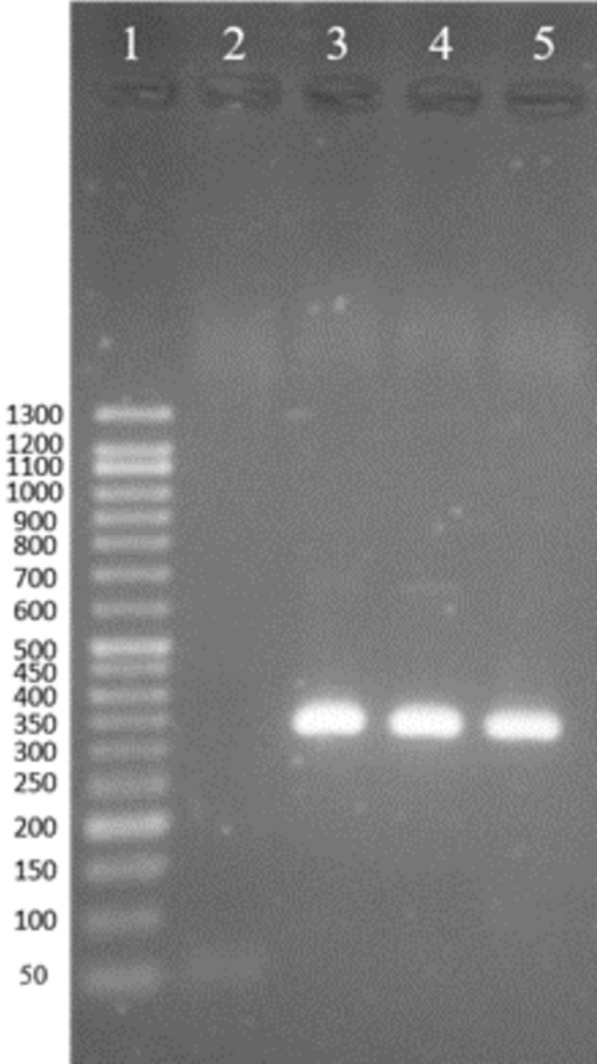
Agarose gel electrophoresis for detection of *Leishmania* analysis after amplification with specific primer pair. Lane 1: 50 DNA ladder, lane 2: negative control (ddH_2_O), lanes 3 and 4: samples detected as *Leishmania*, lane 5: positive control with *L. major* (MRHO/IR/75/ER). The amplicon with the length of about 300–350 bp was considered *Leishmania* spp

**Fig. 4:**
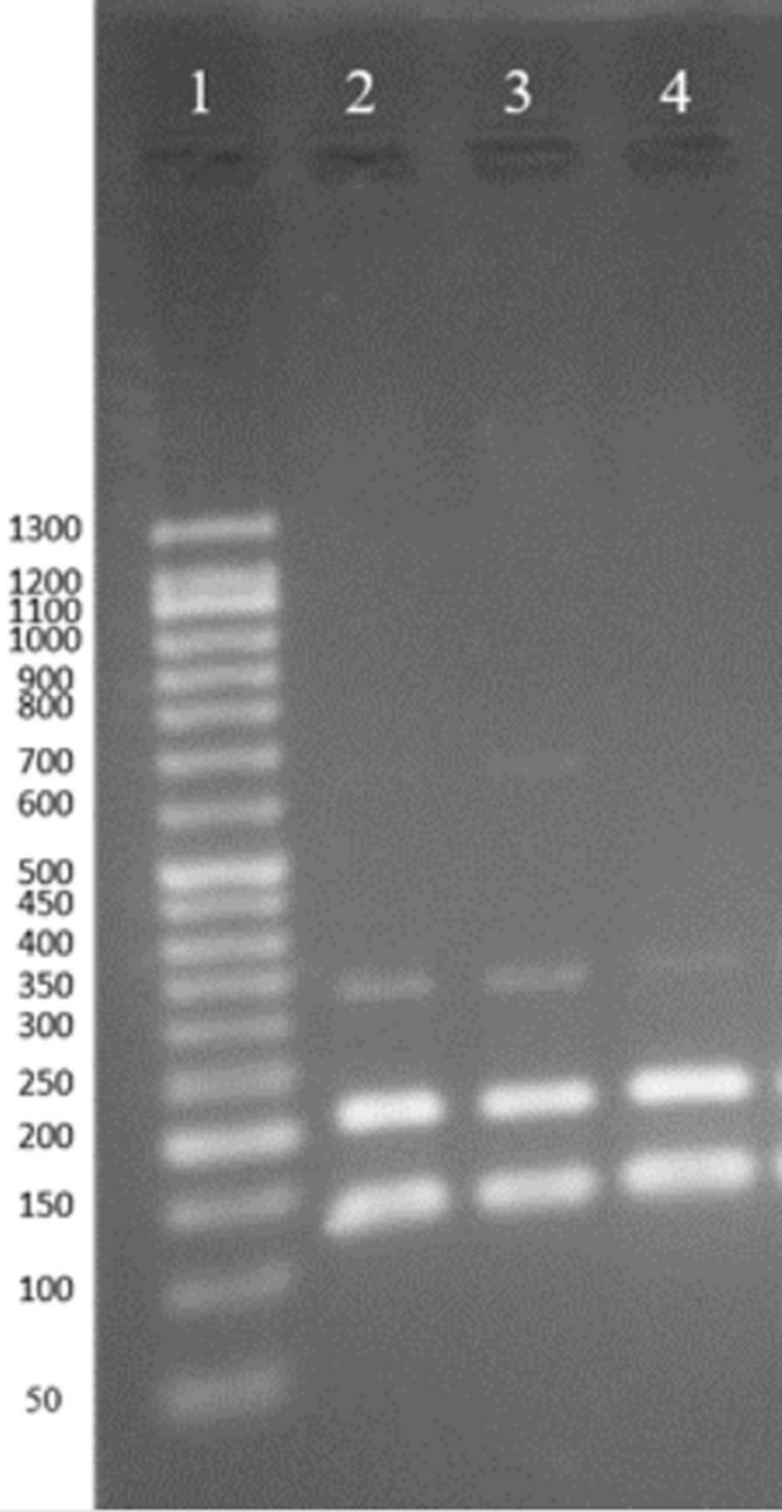
Agarose gel electrophoresis for identification after RFLP analysis. Lane 1: 50 bp DNA ladder, lanes 2 and 3: samples identified as *L. major*, lane 4: standard strain of *L. major* (MRHO/IR75/ER). The fragments with the sizes of 220 and 127 bp was considered *L. major*

**Fig. 5:**
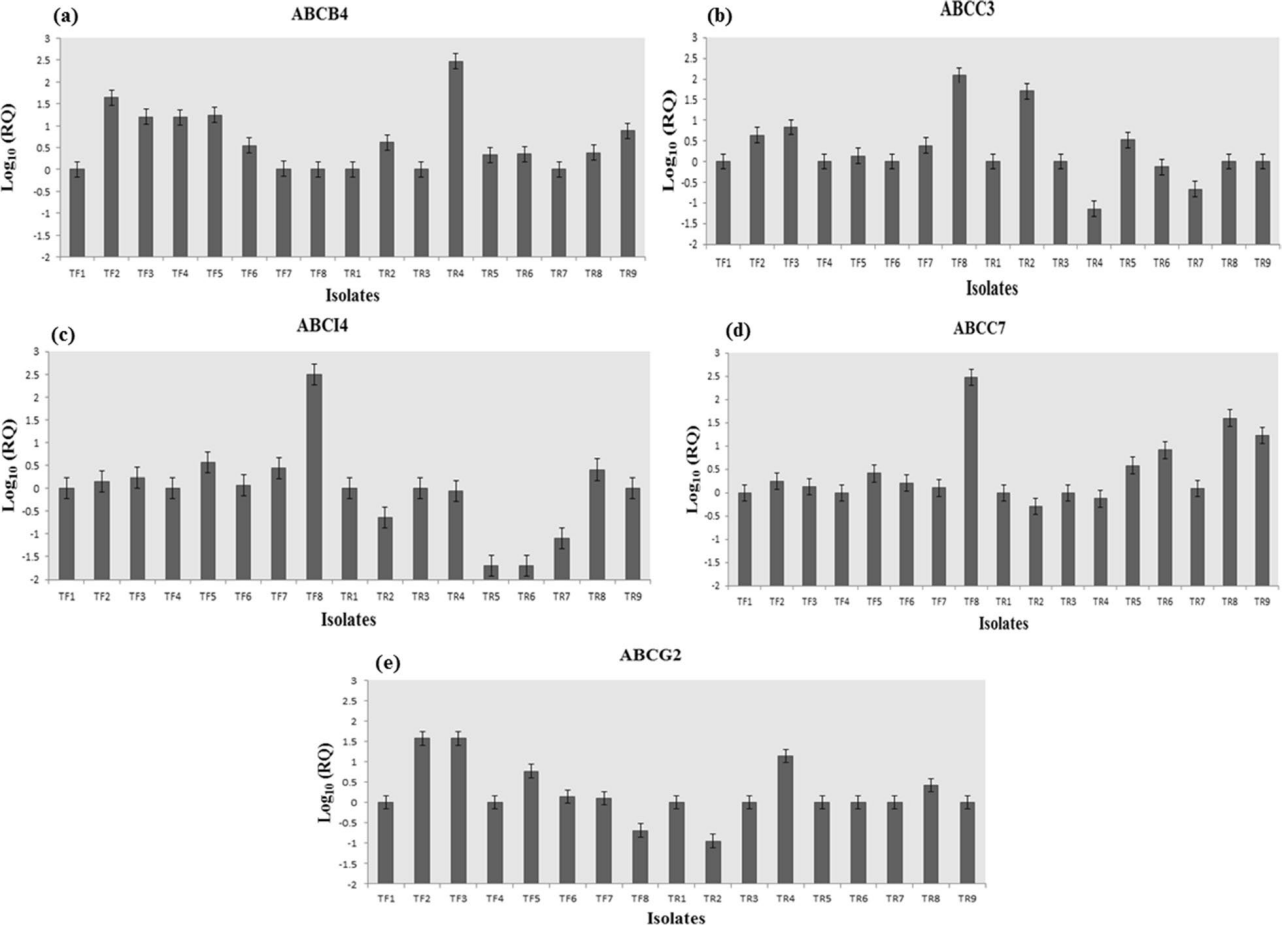
Relative gene expression (Log_10_ RQ) of **a** *ABCB4*, **b** *ABCC3*, **c** *ABCI4*, **d** *ABCC7*, **e** *ABCG2* in clinical isolates of *Leishmania major* by real-time PCR. Isolates TF1-TF8 showed failure response; isolates TR1-TR9 were drug sensitive

All isolates were verified as *Leishmania* using ITS1-PCR with a fragment of about 300–350 bp after amplification (Fig. 3). RFLP analysis showed 220 and 127 bp fragments identified *L. major* (Fig. 4). The fold changes of *ABCC3, ABCC7, ABCI4, ABCB4*, and *ABCG2* genes in the studied isolates are shown in Fig. 5. The variation was observed in treatment response isolates and only the gene expression pattern for *ABCB4* in all of the TR isolates were overexpression. In total 17 isolates were investigated in this study, 4 isolates were NA (No amplification) for *ABCB4* gene. Among 13 remained cases, all of the TR (7/9) and TF (6/8) isolates showed up regulation. *MDR1* gene was over-expresses as 1.04 to 44.63 folds and 2.17 to 298.1 folds in TF and TR isolates, respectively. The lowest gene expression was observed in response to the treatment group (TR5 and TR6 isolates) for *ABCI4* gene. The isolate TF8 for *ABCI4* and *ABCC7* gene showed highest expression with 362.89 and 303.61 fold more gene expression than the other, respectively.

## Discussion

Despite some complications, pentavalent antimony compounds are the recommended first line drug for treatment of leishmaniasis. However, no response to drug is the major clinical concerns for the control of this infectious disease and underlying mechanism has not been fully determined (Hadighi et al. 2006; Croft et al. 2006). Assessment of members of ATP-binding cassette (ABC) transporter family is necessary to find solution and the aim of present study is to evaluate the expression of *ABCI4*, *ABCG2*, *ABCC7*, *ABCB4*, and *ABCC3* genes in *Leishmania* isolates from patients.

In this study, the gene expression pattern for *ABCI4* and *ABCC7* was the same in treatment failure isolates with overexpression in all isolates except TF1 and TF4. *ABCI4* in most of the isolates which were susceptible to drug response (5 of 8), had down regulation. Also, two of the TR cases (TR2 and TR4) had *ABCC7* and *ABCI4* low expression. In a study completed by Manzano et al. (2013) strains with high *ABCI4* expression had no response to antimonials, pentamidine, and amphotericin B. Given that this transporter remains unknown compared to other ABC transporters, it is expected that the expression of *ABCI4* transporter gene is different in various strains, which might explain possible reasons for the discrepancy with the results of the current study.

The *ABCI4* found in the plasma membrane and in the mitochondria should be homodimerization to be functional. It involves in responding to SbV and SbIII treatment using the efflux of metal-conjugated thiols (Castanys-Munoz et al. 2008). Therefore, the overexpression of *ABCI4* results in treatment failure with antimony (Manzano et al. 2014). In our study, TF8 isolate was the case with overexpression of *ABCI4* and *ABCC7* more than 300-fold compared with the treatment response isolates. It seems that overexpression of *ABCI4* in treatment failure isolates especially TF8 could be considered as one of the important mechanisms to cause treatment failure. Also, various studies on different species and strains of *Leishmania* have shown that the *ABCC7* gene is overexpressed in no response to antimony of *Leishmania* strains (Coelho et al. 2003). In a study by Leprohon et al. (2009), it was concluded that ABCC7, ABCC4, and ABCC5 were located in a tubular enclosure along the longitudinal axis of the parasite. Overexpression of at least four members, ABCC3, ABCC4, ABCC5, and ABCC3 can also result in no response to treatment.

The results of our study showed the overexpression pattern of *ABCG2* in all treatment failure isolates except TF8. Perea et al. (2016) concluded that parasites, which overexpress *ABCG2*, have no response to antimony due to reduced SbIII accumulation and increase drug flow. Additionally, ABCG2 could transfer thiol in the presence of SbIII. Rugani et al. (2019) reported that *ABCG2* has no association with respond to treatment in *L. braziliensis* clinical isolates that confer with the results of our study for TF8. This mentioned isolate had *ABCI4*, *ABCC3*, and *ABCC7* over expression (362, 122, and 303-fold, respectively) that may involve in treatment failure response.

*ABCC3* had overexpression in all treatment failure isolates besides TF1, TF4, and TF6 that had no amplification. ABCC3 is located in the intracellular vesicular membrane and is reported to be involved in no response to arsenite and antimony in *Leishmania* promastigotes and amastigotes (Légaré et al. 2001). Coinciding with this study, in El Fadili et al. (2005) study, the expression of the *ABCC3* transporter was continuously increased with mediated by *ABCC3* that can be reversed using the butyrin sulfoximine inhibitor for glutathione biosynthesis. In a study by Jabini et al. (2015), it was reported that silymarin with glucantime may have beneficial effects in models of leishmaniasis mice with no response to drug. Although this study did not show a significant effect on the combined use of silymarin and glucantime, the reason for such a result could be the involvement of other factors and transmitters in response to treatment. Due to the high expression of this transmitter and the emphasis on its importance in the treatment outcomes of leishmaniasis in previous studies (Mukherjee et al. 2007; Légaré et al. 2001; Callahan et al. 1994), in our study, MRPA was considered as one of the potential factors in causing non-response to the treatment of leishmaniasis patients. Other genes may affect the expression of genes under study and complicate no response to treatment. For example, the increased expression of ubiquitin and amino acid permease (AAP3) genes can increase the activity of multidrug-resistance protein A or ABCC3 (Fekrisoofiabadi et al. 2019; Kazemi-Rad et al. 2013).

*MDR1* gene expression seems to be effective on drug response in *Leishmania* spp. in our study, *MDR1* gene had overexpression in TF and TR isolates. In a study carried out by Purkait et al. (2012), the expression level of *MDR1* is found to be higher in the no response to treatment strain, suggesting a higher rate of efflux of amphotericin B.

In conclusion, treatment failure in *Leishmania* spp. represents an intrinsic feature of the parasite that is an adaptive trait. Exposure of the parasite with insufficient doses resulted in expression of ATP-binding cassette (ABC) transporters that induce occurrence of this phenotype that exhibits in the patient, although it is a multifactorial event. In this study we showed that the relative expression of *ABCB4, ABCC3, ABCC7, ABCG2*, and *ABCI4* genes in *L. major* isolates with no response to treatment may be effective as one of the active mechanisms in response to treatment. Since there is a variety of expression among the isolates, it is recommended to investigate the proteins modulating the expression level ABC transporters in next studies.

## Data Availability

The datasets used and analyzed during the current study are available from the corresponding author on reasonable request.

## Abbreviations

ABC: ATP-binding cassette transporters
TF: Treatment failure
TR: Treatment response
CL: Cutaneous leishmaniasis
VL: Visceral leishmaniasis
MCL: Muco-Cutaneous Leishmaniasis
PGPA: P-glycoprotein A
AQP: Aquaglyceroporin
MRP: Multidrug resistance protein
ITS: Internal Transcribed Spacer
RFLP: Restriction Fragment Length Polymorphism
